# Response: Commentary: Long-term Practice with Domain-Specific Task Constraints Influences Perceptual Skills

**DOI:** 10.3389/fpsyg.2019.00085

**Published:** 2019-01-29

**Authors:** Luca Oppici, Derek Panchuk, Fabio Rubens Serpiello, Damian Farrow

**Affiliations:** ^1^Institute for Health and Sport, Victoria University, Melbourne, VIC, Australia; ^2^Movement Science, Australian Institute of Sport, Canberra, ACT, Australia

**Keywords:** transfer, representative design, futsal, soccer, talent development

We are glad that our original article “Long-term practice with domain-specific task constraints influences perceptual skills” (Oppici et al., 2017) has sparked an interesting debate in the skill acquisition field. Yiannaki et al. (2018) recently published a commentary on our article discussing the results in relation to skill transfer from futsal to football, raising questions about the experimental task adopted, and providing recommendations for future research on this topic. In this reply to their commentary, we clarify some aspects that the authors may have misinterpreted and provide our comments in relation to the examination of skill transfer.

First, it is critical to place our original study in the right context to meaningfully interpret its results. In this study, we did not examine skill transfer from futsal to soccer but, instead, how practicing the passing skill in futsal or soccer influences the development of perceptual behavior underpinning passing. While it is known that skill *similarities* promote skill transfer (Magill, 2011) and Yiannaki et al. suggest measuring futsal-soccer similarities to infer skill transfer, we did not evaluate and had no interest in evaluating how futsal-soccer skill *similarities* may promote skill transfer between the two sports. On the contrary, following the constraints-led approach (Newell, 1986; Davids et al., 2008 [see also Higgins, 1977 for a different perspective on constraints and human behavior]), we evaluated how *differences* in behavior emerge when individuals practice a skill with different task constraints. Therefore, our study should be considered from this perspective, and interpretations on how our study provided preliminary evidence on skill transfer are rather speculative and should be considered with caution. On this, Yiannaki et al. stated “additional research is needed before these findings can be used to inform the potential use of futsal as a skill development tool for 11-aside soccer” (Yiannaki et al., 2018). We could not agree more with this statement, considering that we did not assess and present any results concerning skill transfer (for studies examining skill transfer from futsal to soccer see Oppici et al., 2018a,b).

Second, a clear understanding of the representative design concept, and a clear differentiation between representative design and ecological validity can clarify some of the issues that Yiannaki et al. raised regarding the experimental task we designed. The authors argued that our experimental task (i.e., 5 vs. 5 + goalkeeper game) was not representative because the number of players was different to the “real” game and some rules, such as the offside rule, were excluded. However, a task does not necessarily need to fully replicate an environment (e.g., a soccer game) to be representative of a skill performed in that environment. In this sense, representative (experimental) design refers to the sampling of key constraints in an experiment so that they represent the behavioral setting to which an observed behavior is intended to be generalized (Brunswik, 1955; Dhami et al., 2004). Germane to the investigation of how perception supports action (as our study purported to do), the informational constraints that guide the self-organization of a movement (e.g., the passing action) are the key constraints that need to be represented (Pinder et al., 2011; Davids et al., 2012). In the context of passing, informational constraints emerge from the interaction of a passer with their teammates and opponents, and between teammates and opponents (Travassos et al., 2012; Corrêa et al., 2014). Our 5 vs. 5 + goalkeeper task included the key constraints—opponents and teammates—that shape the emergence of passing information during games, and we have used the average individual playing area that typically occurs in the two sports to differentiate the futsal and soccer tasks. Therefore, our task can be considered representative of short passing skill in the two sports.

Furthermore, Yiannaki et al. suggested the use of “authentic” futsal and soccer games as experimental contexts to increase the ecological validity and, in turn, generalization of the results to the two sports. While agreeing that future research should improve the generalizability of results to futsal and soccer, we argue that ecological validity is not the key for improving the generalization of the results of an experiment but, instead, it is the representative design. Ecological validity is the relation between a perceptual variable and a distal criterion state in the environment (Brunswik, 1956). The ecological validity of a ball or a teammate does not improve from a modified 5 vs. 5 to an 11 vs. 11 soccer game. In both contexts, a player can infer what the ball affords (in terms of possible actions) from the direct perception of the ball. As previously stated, representative design is concerned with the generalization of results (for a thorough explanation of how these two concepts differ see Araújo et al., 2007). Therefore, future research should carefully sample the constraints toward which a player's behavior is intended to be generalized. Given that sampling constraints in their entirety is rarely achievable when assessing an athlete's perception (Mann and Savelsbergh, 2015), future research should clearly acknowledge what constraints an observed behavior can be generalized to (for an example see Oppici et al., 2018a).

Lastly, we must clarify that futsal is not simply a small-sided soccer game (as Yiannaki et al. stated) and we provide recommendations for future research on skill transfer. Various elements, such as the futsal ball, the playing surface and the substitution rule differentiate futsal and soccer and make futsal unique. Therefore, futsal needs to be considered in its entirety when discussed as a developmental activity for promoting talent development in soccer. In regard to future research, we agree with the authors that further research is needed to better clarify skill transfer. However, we do not entirely agree with the authors' suggestions on how to evaluate transfer, i.e., using performance analysis and measuring the qualitative perception of stakeholders. Certainly, performance analysis allows the measurement of a player's behavior in their typical environment (i.e., the game); however, a comparison of soccer and futsal would not evaluate whether a player can transfer their skill to the other sport. Transfer is evaluated on performance achievement (Araújo and Davids, 2015), and futsal players have to perform a task in soccer to assess whether their previous experience in futsal promotes behavior functionality (thus positive transfer) in soccer. The design of an appropriate soccer task will create some challenges (we provided an option) but it essential for the evaluation of skill transfer. Related to how transfer is evaluated, it is unclear how qualitative measures, as suggested by the authors, can provide evidence of skill transfer. In conclusion, we appreciate the Yiannaki et al. commentary, as a constructive debate can improve the examination of hot topics like the transfer of skill from futsal to football. However, a number of commentaries have now been published on this topic and we look forward to seeing new empirical evidence that clarifies how skill may transfer from futsal to soccer.

## Author Contributions

All authors listed have made a substantial, direct and intellectual contribution to the work, and approved it for publication.

### Conflict of Interest Statement

The authors declare that the research was conducted in the absence of any commercial or financial relationships that could be construed as a potential conflict of interest.
